# Numerical Study on the Surface Plasmon Resonance Tunability of Spherical and Non-Spherical Core-Shell Dimer Nanostructures

**DOI:** 10.3390/nano11071728

**Published:** 2021-06-30

**Authors:** Joshua Fernandes, Sangmo Kang

**Affiliations:** Department of Mechanical Engineering, Dong-A University, Busan 49315, Korea; joshuafernandes95@gmail.com

**Keywords:** electromagnetic field enhancement, localized surface plasmon resonance, plasmonic nanostructures, aspect ratio, refractive index, shell thickness, distance of separation

## Abstract

The near-field enhancement and localized surface plasmon resonance (LSPR) on the core-shell noble metal nanostructure surfaces are widely studied for various biomedical applications. However, the study of the optical properties of new plasmonic non-spherical nanostructures is less explored. This numerical study quantifies the optical properties of spherical and non-spherical (prolate and oblate) dimer nanostructures by introducing finite element modelling in COMSOL Multiphysics. The surface plasmon resonance peaks of gold nanostructures should be understood and controlled for use in biological applications such as photothermal therapy and drug delivery. In this study, we find that non-spherical prolate and oblate gold dimers give excellent tunability in a wide range of biological windows. The electromagnetic field enhancement and surface plasmon resonance peak can be tuned by varying the aspect ratio of non-spherical nanostructures, the refractive index of the surrounding medium, shell thickness, and the distance of separation between nanostructures. The absorption spectra exhibit considerably greater dependency on the aspect ratio and refractive index than the shell thickness and separation distance. These results may be essential for applying the spherical and non-spherical nanostructures to various absorption-based applications.

## 1. Introduction

Plasmonic core-shell nanoparticles are one of the most powerful optical and thermal contrast agents in the field of biomedicine [1,2,3,4] with a lot of attention drawn towards cancer treatment [5,6,7]. The strong interaction of these metal nanoparticles (MNPs), with the incident electromagnetic field allows them to exhibit unique optical properties [8]. The collective free electron oscillation in the nanoparticles gives rise to the localized surface plasmon resonance (LSPR) effect, which can result in the enhancement of absorption and scattering of the incident electromagnetic waves [9,10,11,12,13]. Gold nanoparticles have gained a lot of attention because of their strong optical absorption capacity and high resonance wavelength tunability [14]. The maximization of near-field enhancement in the gold nanoparticles depends strongly on the geometrical aspects, dielectric properties, and compositional factors [15,16,17,18,19]. Small differences in the geometrical features will lead to shifts in the optical properties of these nanoparticles, and the resulting resonance wavelengths can span from visible to near-infrared (NIR) regions. In order to obtain deeper penetration of light into the human tissues, ideal nanomaterials that can strongly absorb energy in the optical window have to be developed.

In recent years, the LSPR characteristics of nanospheres and nanorods, among several other nanostructures, have been studied [20,21,22]. Researchers have successfully used the Mie theory and discrete dipole approximation (DDA) methods to investigate the optical properties of different gold nanostructures [7]. Extensive Mie theory calculations reveal that the plasmon resonance wavelength maximum shifts almost exponentially as we move from a solid gold nanosphere (AuNP) to a gold nanoshell (AuNS) with decreasing shell thickness [23]. Among all gold nanoparticles, the ultra-thin hollow gold nanoshells (AuNSs) produced by the synthesis of cobalt (Co) nanoparticles as sacrificial templates induce a unique tunable absorption band in the near-infrared tissue window of 650–950 nm (NIR-I window), where tissues are maximally transparent [24,25,26,27]. AuNSs demonstrating plasmon resonance in this tissue window, exhibit strong absorption and scattering characteristics depending on their morphology and can be useful bioimaging and photothermal ablation agents [28,29]. The gold shell filled with the aqueous embedding medium should be large enough to allow prolonged blood circulation time for biomedical applications [30,31,32].

Upon coupling of two identical core-shell nanoparticles to form a dimer, plasmonic modes with high field enhancement in the gap of nanoshells occur due to the dipolar mode coupling of the individual particles [33]. Due to the occurrence of an extremely intense electric field confinement in the nanogap, the dimer structures are drawing attention in the optical techniques. Experimental and theoretical advances are currently being made in the field of plasmonics, specifically the near-field enhancement in core-shell dimer nanostructures. The low energy absorption and minimal scattering in the NIR-II window (1000–1700 nm) provide better tissue transmission rate and lower photon scattering for the incident light, which is highly suitable for biomedical applications and photoacoustic imaging [34,35,36]. Compared to the NIR-I window, the larger penetration depth and higher maximum permissible exposure (MPE) to the laser are clear advantages of working in the NIR-II window. However, very few studies have been performed in the NIR-II window because of the lack of photosensitive materials of morphology needed to attain high photothermal efficiency in this tissue window [37,38]. It is noteworthy that no numerical investigation of the plasmonic properties of non-spherical dimer nanostructures is explored. This motivates us to investigate models of prolate and oblate-shaped non-spherical dimer nanostructures to determine their LSPR peaks and absorption cross-sections. Thus, the present study aims at scrutinizing aspects affecting the resonance frequency and enhancement factor for the gold-based nanostructures.

In this study, we numerically study the optical properties of core-shell nanoparticle dimers of spherical and spheroidal (non-spherical) shapes. The finite element modelling study is performed using a commercial software COMSOL Multiphysics to describe the localized surface plasmon resonance behaviour in the region of separation of the dimer nanostructures. This study compares the optical properties obtained using the non-spherical prolate and oblate dimer nanostructures with those of the spherical dimer nanoshells. The effects of varying the aspect ratio, refractive index of the surrounding medium, shell thickness while maintaining a constant outer radius, and distance of separation between nanostructures are studied. The results clearly show that a wide range of tunability of the LSPR peaks in the NIR windows can be obtained for the non-spherical prolate and oblate nanostructures when compared to the spherical nanoshell dimer. The remainder of this study is organized by first including the theoretical equations and numerical method. Secondly, the validation of the numerical method and the effect of the aspect ratio on the LSPR is investigated for the gold nanostructures. Thirdly, the dependence of LSPR on the refractive index of the surrounding medium is studied for the spherical and non-spherical gold nanostructures by using different refractive indices for the surrounding medium. Finally, we solve the absorption cross-section and electromagnetic field enhancement factor by varying the shell thickness and distance of separation between core-shell nanostructures.

## 2. Numerical Method

**Figure 1:**
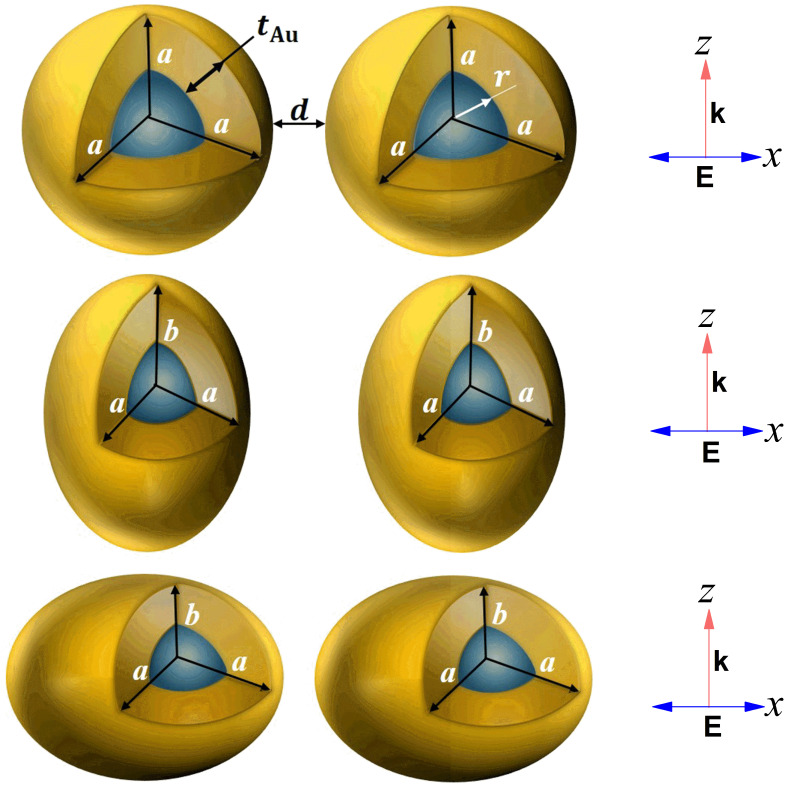
Schematic diagrams of the spherical and non-spherical core-shell nanostructures (top row: spherical, middle: prolate, bottom: oblate). *a* and *b* are the semi-principal axes of the nanostructures, *d* is the distance of separation between the outer surfaces of the nanostructures, $t_{\mathrm{Au}}$ is the Au shell thickness, and *r* is the core radius for the spherical Au nanoshell (AuNS). **k** is the incident plane wave along the *z*-axis, and **E** is the electric field polarization along the *x*-axis.

The numerical study of the optical properties of plasmonic nanostructures using the finite element method (FEM)-based solver is one of the best alternative methods for the complex analytical solution to problems involving multiple nanostructures. The schematic diagrams of core-shell plasmonic dimer nanostructures with a gold shell and a core made of an aqueous medium are depicted in Figure 1. We perform a 3D modelling and simulation study based on FEM using a versatile commercial software package COMSOL Multiphysics. The optical properties of core-shell structured nanoparticles are calculated using the wave optics module in COMSOL Multiphysics. The spherical and non-spherical dimers are illuminated by an incident plane wave propagating along the *z*-axis and the electric field polarization along the *x*-axis with the background electric field$\;E_{b}=E_{0}\exp\left(-jk_{0}z\right)$. Here, $E_{0}$ is the incident electric field, which is set to unity in this numerical study, and $k_{0}$ is the free space wave number. Assuming that the electric field is time harmonic, the electric field distribution E is computed using the Helmholtz equation and represented as follows [39]:

(1) $$\nabla \times \left(\mu_{r}^{-1}\nabla \times E\right)-k_{0}^{2}\left(\varepsilon_{r}-j\frac{\sigma }{\omega \varepsilon_{0}}\right)E=0,$$

where $\mu_{r}$ is the relative permeability, which is taken as unity, ω is the frequency of the incident light, $\varepsilon_{0}$ is the permittivity of free space, σ is the conductivity, and $\varepsilon_{r}$ is the relative permittivity. The relative permittivity, $\varepsilon_{r}=\varepsilon /\varepsilon_{0}\;$, is a frequency-dependent complex dielectric quantity for the gold shell, henceforth symbolized as $\varepsilon_{\mathrm{Au}}$ in this study. Moreover, as the thickness of the thin metal shell becomes comparable to the mean free path of conduction electrons (~42 nm), the size effect of bound electrons and interband transition must also be taken into consideration when calculating the dielectric constant of metal shell [40]. Thus, the complex dielectric function of gold$\;\varepsilon_{\mathrm{Au}}$ is introduced by using the Drude–Lorentz model as follows [41]:

(2) $$\varepsilon_{\mathrm{Au}}=\varepsilon_{\mathrm{Au}-\exp}+\frac{\omega_{p}^{2}}{\omega \left(\omega +i\gamma_{\mathrm{bd}}\right)}-\frac{\omega_{p}^{2}}{\omega \left(\omega +i\left(\gamma_{\mathrm{bd}}+\gamma_{\mathrm{sd}}\right)\right)}\;,$$

where $\varepsilon_{\mathrm{Au}-\exp}$ is the bulk dielectric function for gold [42], and $\omega_{p}$, $\;\gamma_{\mathrm{bd}}$, and $\gamma_{\mathrm{sd}}$ are the plasma frequency, bulk damping, and damping due to surface scattering, respectively [41]. The damping due to surface scattering is added to the bulk damping in Equation (2) to obtain the corrected dielectric function for the shell material and is given by [43]:

(3) $$\gamma_{\mathrm{sd}}=\frac{Av_{f}}{l_{\mathrm{eff}}}.$$

Here, the phenomenological dimensionless fitting parameter A ranges from 0 to 1 describing the nature of surface scattering and is taken as unity in all simulations [44]. $v_{f}$ is the Fermi velocity of carrier electrons in the gold shell $\left(1.40\times 10^{6}\;{\mathrm{ms}}^{-1}\right)$, and the effective mean free path of the electrons $l_{\mathrm{eff}}$ in the shell is taken to be equal to the thickness of the gold shell $t_{\mathrm{Au}}$. Perfectly matched layers (PMLs) together with a scattering type boundary condition are used to absorb the waves outgoing from the finite-sized computational field that emulates an infinite domain.

The relative absorption and scattering ability of the core-shell nanostructures can be obtained by defining the absorption and scattering cross-sections,$\;\sigma_{\mathrm{abs}}$ and$\;\sigma_{\mathrm{sca}}$, as follows [45,46]:

(4) $$\sigma_{\mathrm{abs}}=\frac{1}{I_{0}}∭Q_{r}dV,$$

(5) $$\sigma_{\mathrm{sca}}=\frac{1}{I_{0}}\iint \left(n\cdot S_{\mathrm{sca}}\right)dS,$$

where $Q_{r}$ is the power loss density in the nanostructures, **n** denotes the normal vector pointing outward from the surface of the nanostructures, $S_{\mathrm{sca}}$ stands for the Poynting vector $\left({\mathrm{Wm}}^{-2}\right),\;$*V* and *S* are the volume and surface area of the nanostructures, respectively, and $I_{0}=\varepsilon_{0}nE_{0}^{2}/2$ represents the intensity of the incident wave of amplitude $E_{0}$ in the surrounding medium of refractive index *n*. The enhancement of the incident electromagnetic field in the AuNSs by several orders of magnitude, leads to numerous absorption and scattering-based applications, such as surface enhanced Raman scattering [47], tip-enhanced Raman spectroscopy [48], or drug delivery. $I_{0}=\varepsilon_{0}nE_{0}^{2}/2$ indicates that the local field intensity of the incident wave at a specific point is proportional to the square of the electric field amplitude. The electromagnetic local field enhancement factor (LFEF) at a particular position $\left(r_{0}\right)$ in the vicinity of the nanostructures can be given as the ratio of the electric field amplitude at that position $\left(E{\left(r_{0}\right)}^{2}\right)$ to the electric field amplitude of the incident wave$\;\left(E_{0}^{2}\right)$. Taking into account the dependency of LFEF on the frequency $\left(\omega_{0}\right)$ of the incident wave, the LFEF as a function of the position and frequency can be represented as$\;\mathrm{LFEF}\left(r_{0},\omega_{0}\right)$. In addition, the incoming incident wave at $\omega_{0},$ while emitting a scattered photon, causes the strong mutual excitation between the dipoles of molecules and nanostructures at the Raman scattered frequency$\;\left(\omega_{R}\right)$. This gives rise to the local field enhancement at the Raman frequency, and the enhancement factor can be represented as$\;\mathrm{LFEF}\left(r_{0},\omega_{R}\right)$. The total electromagnetic enhancement factor (EF) can be expressed by the fourth power relation and can be given as [49]:

(6) $$\mathrm{EF}=\mathrm{LFEF}\left(r_{0},\omega_{0}\right)\mathrm{LFEF}\left(r_{0},\omega_{R}\right)=\frac{{\left|E\left(r_{0},\omega_{0}\right)\right|}^{2}}{{\left|E_{0}\left(r_{0},\omega_{0}\right)\right|}^{2}}\frac{{\left|E\left(r_{0},\omega_{R}\right)\right|}^{2}}{{\left|E_{0}\left(r_{0},\omega_{R}\right)\right|}^{2}}\approx \frac{{\left|E\left(r_{0},\omega_{R}\right)\right|}^{4}}{{\left|E_{0}\left(r_{0},\omega_{0}\right)\right|}^{4}}\;.$$

Here, the first enhancement is due to the coupling of plasmons and incident photons, and the second enhancement is due to the coupling of plasmons and emitted photons [50,51]. The fourth power relation is obtained by taking into consideration an assumption that the Raman scattered frequency is close enough to the frequency of incident wave$\;\left(\omega_{R}\approx \omega_{0}\right)$.

## 3. Results and Discussion

The optical properties of core-shell nanostructures depend strongly on the size and shape of the nanostructures and their surrounding environment. The spherical, prolate and oblate-shaped nanostructures are numerically simulated to analyse the interaction of the incident light with the dimer configurations. The effects of various core-shell dimer parameters on the optical properties and tuning sensitivity are studied for all three types of nanostructures. The total radius of the spherical AuNS and the effective radius of the non-spherical prolate and oblate nanostructures are kept similar throughout this study for comparisons of the numerically simulated optical properties. The spherical core-shell nanoparticle dimer is composed of an inner core with radius *r* (nm) and a gold shell with the thickness $t_{\mathrm{Au}}$ (nm), having an interparticle distance *d* (nm). The effective radius of the non-spherical prolate and oblate nanostructures is given by the term$\;r_{\mathrm{eff}}=\sqrt[{3}]{abc}$, where *a*, *b*, and *c* are the semi-principal axes of the non-spherical nanostructures. In all simulations, the values of *a* and *c* are kept identical (*a = c*), and the aspect ratios for the non-spherical prolate and oblate structures are defined as η=b/a and η=a/b, respectively. Note that, η=1 represents the case of spherical nanostructures.

### 3.1. Numerical Validation

**Figure 2:**
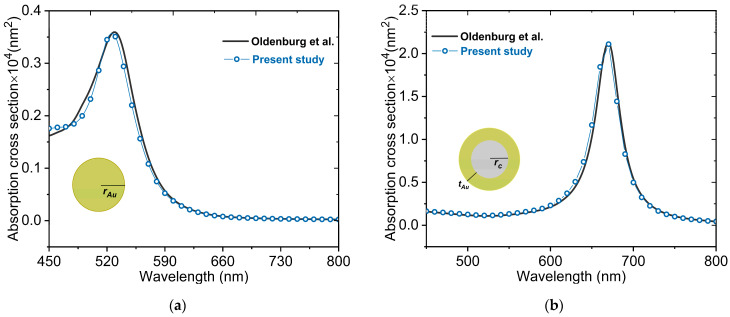
Validation of the present numerical model: Absorption spectra for the (**a**) solid gold nanosphere (AuNP) $\left(r_{\mathrm{Au}}=20\;\mathrm{nm}\right)\;$ and (**b**) silica-gold core-shell nanoshell $\left(r_{c}=20\;\mathrm{nm},\;t_{\mathrm{Au}}=3\;\mathrm{nm}\right)$ as a function of the incident wavelength, compared to the Mie theory [52]. The nanostructures are surrounded by water with a refractive index of 1.33.

In order to validate our numerical approach that consists mainly of FEM using COMSOL Multiphysics package (Version 5.6, COMSOL, Inc. Burlington, MA), we simulate two cases, AuNP and AuNS, whose absorption characteristics are available in the literature [52]. The absorption cross-sections for a AuNP and a AuNS for the wavelength range of 450–800 nm are depicted in Figure 2a,b, respectively. The present numerical approach is validated by comparing the numerically obtained absorption cross-sections with those of Oldenburg et al. [52] using the Mie theory. Our numerical results for both nanostructures are in excellent agreement with the theoretical results derived using the Mie theory, indicating that our numerical investigation is accurate enough for the purpose of this study.

### 3.2. Mesh Independence Test

**Figure 3:**
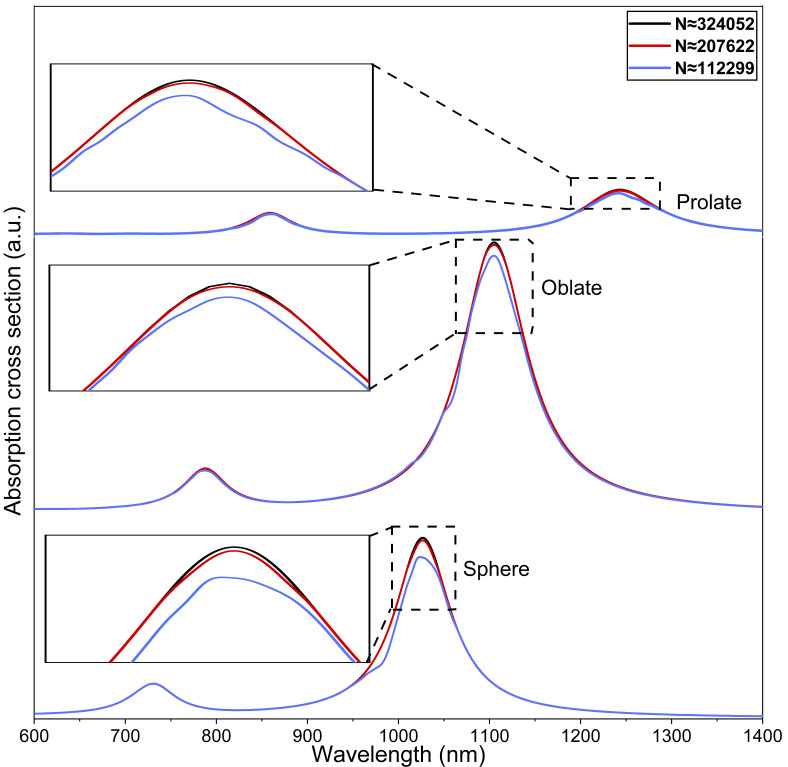
Mesh independence test: Absorption cross sections for the spherical $\left(r=20\;\mathrm{nm},\;t_{\mathrm{Au}}=3\;\mathrm{nm}\right)\;$ and non-spherical$\;\left(r_{\mathrm{eff}}=23\;\mathrm{nm},\;\eta =3\right)$ core-shell nanostructures. The nanostructures are surrounded by water with a refractive index of 1.33. Here, N is the total number of mesh elements used in the present study.

A mesh independence test with identical physical and optical parameters but with a variable number of mesh elements is performed to establish the accuracy of our numerical study. A tetrahedral meshing scheme is adopted for the modelling of the spherical and non-spherical nanostructures. The PMLs with a spherical type swept mesh having 5 elements across the diameter are used to reduce the reflections in the interior, which then provides a better convergence of the solver and maximizes the absorption of the propagating wave. For an increase in the total number of elements from N ≈ 207622 to ≈324052, the maximum change in the LSPR peak is 0.01%, as shown in Figure 3. By taking into consideration the computational cost and independence of mesh, the mesh comparable qualitatively to N ≈ 207622 is employed for all numerical simulations performed in this study.

### 3.3. Aspect Ratio of Prolate and Oblate Nanostructures

**Figure 4:**
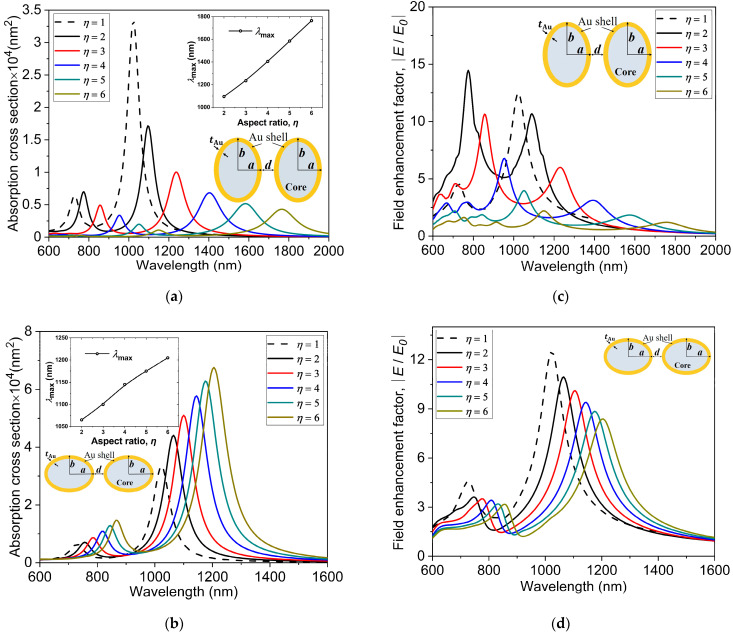
Absorption cross-sections at different aspect ratios for the (**a**) prolate and (**b**) oblate core-shell nanostructures$\;\left(r_{\mathrm{eff}}=23\;\mathrm{nm},\;n=1.33\right)$. The insets in panels (**a**) and (**b**) show the dependence of the LSPR peak position $\left(\lambda_{\max}\right)$ on the aspect ratio. The electric field enhancement $\left|E/E_{0}\right|$ at different aspect ratios for the (**c**) prolate and (**d**) oblate nanostructures. The absorption cross-sections and electric field enhancement for the spherical core-shell dimer nanostructures $\left(\eta =1\right)$ are also shown for comparison (black dashed curves).

The spectral characteristics of the spheroidal core-shell nanostructures with prolate and oblate shapes having an effective radius of $r_{\mathrm{eff}}=23\;\mathrm{nm}$ and a shell thickness of $t_{\mathrm{Au}}=3\;\mathrm{nm}$ for different aspect ratios are shown in Figure 4. The core is considered to be made of an aqueous medium with a refractive index of 1.33. The longitudinal polarization of light for strongly interacting dimers is considered for both nanostructures, as shown in Figure 4a,b. As can be seen in Figure 4a, the maximum absorption cross-section for the prolate spheroid nanostructure is $1.71\times 10^{4}\;{\mathrm{nm}}^{2}$ at an aspect ratio η=2. The corresponding peak resonance wavelength for this absorption peak is 1095 nm, which occurs in the lower wavelength range of the NIR-II window. However, as η increases from 2 to 6, a significant increase in the amount of red-shift is observed in the absorption spectrum. The wavelength corresponding to the peak resonance at η=6 is located near 1765 nm, which is away and beyond the NIR-II window. This implies that for the prolate structure, the LSPR peak can be rigorously red-shifted, specifically providing an extensive range of tunability in the NIR-II window by changing the aspect ratio. For the sake of comparison between the spherical and non-spherical Au nanostructures, the absorption cross-section for the spherical AuNS dimer$\;\left(\eta =1\right)$ is also shown in Figure 4a,b (black dashed curves). It is noteworthy that the intensity of the LSPR peak for the spherical AuNS dimer is higher than that for the prolate dimer nanostructure. However, it is clear from Figure 4a that the position of the LSPR peak for the prolate dimer exhibits excellent tunability in the NIR windows. In addition, the occurrence of plasmon modes in the shorter wavelength range is primarily due to the dipolar plasmon resonances and close proximity of the two prolate nanostructures.

Figure 4b shows the absorption cross-sections at different aspect ratios for the oblate spheroid. The amount of red-shift in the LSPR position in the longer wavelength range is much smaller than that for the prolate dimer nanostructure. However, as the aspect ratio increases, the peak of absorption cross-section is found to remarkably increase, which is opposite to the absorption cross-section response for the prolate dimer nanostructures. The LSPR peak for the aspect ratio η=6 is 1.5 times the value obtained for η=2 (the corresponding wavelengths are 1205 nm for η=6 and 1065 nm for η=2). Although the range of the absorption spectrum can be tuned to a certain extent in the NIR-II window for the oblate dimer nanostructures, it is not as wide as the range of the prolate dimer nanostructures for the same aspect ratios. The insets in the two panels (a) and (b) display the variations of the wavelength corresponding to the LSPR peak with the aspect ratio for the spheroidal nanostructures. The electric field enhancements, $\left|E/E_{0}\right|$, at different aspect ratios are presented in Figure 4c,d for the prolate and oblate nanostructures, respectively. A clear red-shift in $\left|E/E_{0}\right|$ on the surface of prolate and oblate nanostructures together with a decrease in magnitudes can be seen in the panels. The peaks of near-field enhancement are distributed across a wide wavelength range, with the peak values ranging extensively from 1.2 to 14.5 in the two biological windows (NIR-I and NIR-II).

### 3.4. Refractive Index of the Surrounding Medium

The absorption spectrum of a gold-based nanostructure depends on numerous parameters such as the morphology of nanostructures, incident electromagnetic field, and refractive index (*n*) of the surrounding medium [53]. The refractive index of the surrounding medium is one of the main factors on which the sensitivity of the LSPR depends. In this subsection, we investigate the effect of varying the refractive index, n, on the absorption spectrum to clearly understand the surrounding medium-dependent optical characteristics of gold nanostructures of spherical and non-spherical shapes. The two spherical AuNSs $\left(r=20\;\mathrm{nm},\;t_{\mathrm{Au}}=3\;\mathrm{nm}\right)$ are separated by a distance of d=1 nm (the distances between the outer surfaces of the nanostructures) in all simulations. In order to compare the absorption spectrum of the spherical AuNS dimer to the non-spherical prolate and oblate dimer nanostructures, the total effective radii $\left(r_{\mathrm{eff}}\right)$ of the non-spherical nanostructures are kept the same as the total radius of the spherical AuNS, together with an aspect ratio of η=3.

**Figure 5:**
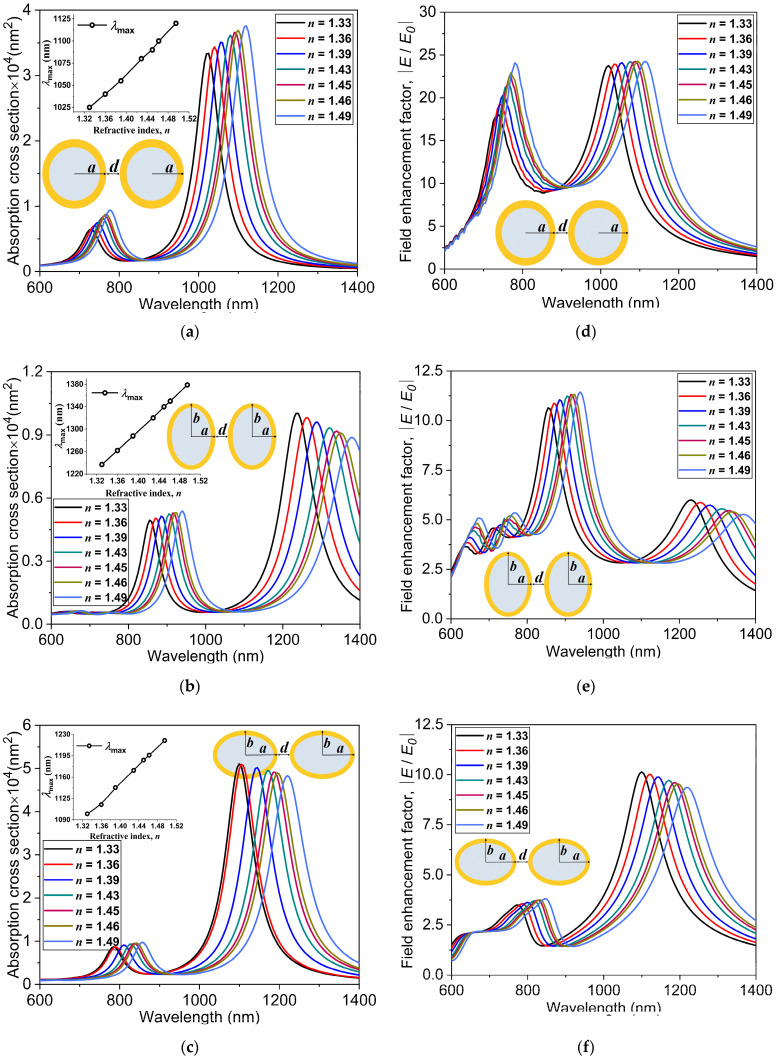
Absorption cross-sections at different refractive indices of the surrounding medium for the (**a**) spherical $\left(r=20\;\mathrm{nm},\;t_{\mathrm{Au}}=3\;\mathrm{nm}\right)$, (**b**) prolate and (**c**) oblate core-shell nanostructures$\;\left(r_{\mathrm{eff}}=23\;\mathrm{nm},\;\eta =3\right)$. The insets in panels (**a**–**c**) show the dependence of the LSPR peak positions $\left(\lambda_{\max}\right)$ on the refractive index. The electric field enhancement $\left|E/E_{0}\right|$ at different refractive indices for the (**d**) spherical, (**e**) prolate, and (**f**) oblate core-shell nanostructures.

To understand the sensitivity of absorption spectra of the spherical and non-spherical nanostructures to the change in the refractive index of the surrounding medium, we perform a set of numerical simulations by placing the nanostructures in solvents of different refractive indices. As shown in Figure 5a, as the refractive index increases from n=1.33 (water) to 1.49 (toluene), a clear red-shift in the LSPR peak for the spherical AuNS dimer is observed. The lower energy plasmon modes occurring in the longer wavelength region (NIR-II window) and the higher energy secondary modes occurring in the shorter wavelength region (NIR-I window) indicate that they can be easily tuned in the two biological windows by varying the refractive index of the solvent. Similar kinds of absorption spectra can be observed for the non-spherical prolate and oblate nanostructures, as depicted in Figure 5b,c respectively. Besides, the amount of red-shift is significantly greater for the non-spherical nanostructures as opposed to the spherical nanostructure. The wavelengths corresponding to the LSPR positions at *n* = 1.49 for the spherical, oblate, and prolate nanostructures are 1100 nm, 1220 nm, and 1380 nm, respectively. The plasmon resonance peaks and their positions for these spherical and non-spherical gold nanostructures exhibit great dependency on the real part of the dielectric constant of the metal, ReεAu [54]. The spectral position of the absorption peak is represented by the following resonance condition [44]:

(7) ReεAu≈−2εm,

where $\varepsilon_{m}$ is the dielectric constant of the surrounding medium, which is equal to$\;n^{2}$ [55,56,57,58]. This dependency on the refractive index is clearly evident in the absorption spectra obtained for the spherical, prolate, and oblate nanostructures, as can be seen in Figure 5a–c. The insets in Figure 5a–c depict the variations of the LSPR peak with the refractive index.

**Figure 6:**
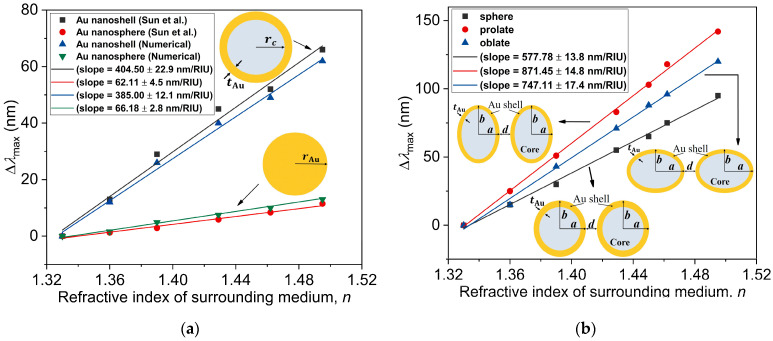
(**a**) Variations of the relative shift in the peak position (with respect to that for water, *n* = 1.33), $\Delta \lambda_{\max}$, with the refractive index of the surrounding medium for the Au nanoshell $\left(r_{c}=25\;\mathrm{nm},t_{\mathrm{Au}}=4.5\;\mathrm{nm}\right)$ and solid Au nanosphere $\left(r_{\mathrm{Au}}=25\;\mathrm{nm}\right)$, compared with the experimental study of Sun et al. [59]. (**b**) Variations of the relative shift in the peak position,$\;\Delta \lambda_{\max}$, with the refractive index for the spherical $\left(r=20\;\mathrm{nm},\;t_{\mathrm{Au}}=3\;\mathrm{nm}\right)$ and non-spherical$\;\left(r_{\mathrm{eff}}=23\;\mathrm{nm},\;\eta =3\right)$ core-shell Au nanostructures. The straight lines in panels (**a**) and (**b**) represent the slopes of the data for the nanostructures.

In order to compare our numerical results with experimentally obtained ones, we perform numerical simulations of the hollow AuNS monomer and solid gold nanosphere monomer with the same dimensions as the experimental study of Sun et al. [59]. Their comparison is presented in Figure 6a. The relative shift in the peak position,$\;\Delta \lambda_{\max}$, shows linear dependency on the refractive index of the surrounding medium. Here, $\Delta \lambda_{\max}$ is defined as the relative shift in the peak position from the value of gold nanostructure in water, of *n* = 1.33, to indicate its dependency on the refractive index. The nature of the graph shows that the sensitivity factor is a ratio between the relative shift in the peak position,$\;\Delta \lambda_{\max}$, and the change in the refractive index, Δn (RIU). The sensitivity factors $\left(\Delta \lambda_{\max}/\Delta n\right)$ given by the slopes in this figure are calculated as 385 nm/RIU and 66 nm/RIU for the AuNS and solid Au nanosphere, respectively. These numerically obtained sensitivity factors are in good agreement with the experimental values, 405 nm/RIU and 62 nm/RIU [59]. In the same manner, the sensitivity factors for the numerically simulated spherical and non-spherical Au nanostructures are computed and shown in Figure 6b. The relative shift in the peak position for the dimer nanostructures clearly shows better sensitivity when compared with the monomer counterparts of solid Au nanosphere and AuNS. The slopes for the three dimer nanostructures indicate that the prolate spheroid dimers are the most sensitive nanostructures among the three types, with a slope of 871.45 nm/RIU. This suggests that a wide range of tunability of LSPR in the NIR-II biological window can be obtained for prolate spheroid dimers with a sensitivity 1.5 times more than the case of the spherical AuNS dimers (577.78 nm/RIU). For different surrounding media considered in this numerical study, the electric field enhancements, $\left|E/E_{0}\right|$, for the spherical, prolate, and oblate Au nanostructure dimers are depicted in Figure 5d–f, respectively. It is noteworthy that as the refractive index increases, the maximum of $\left|E/E_{0}\right|$ in the lower energy mode slightly decreases for the non-spherical nanostructures but remains nearly constant for the spherical AuNS dimer.

### 3.5. Au Shell Thickness and Distance of Separation

**Figure 7:**
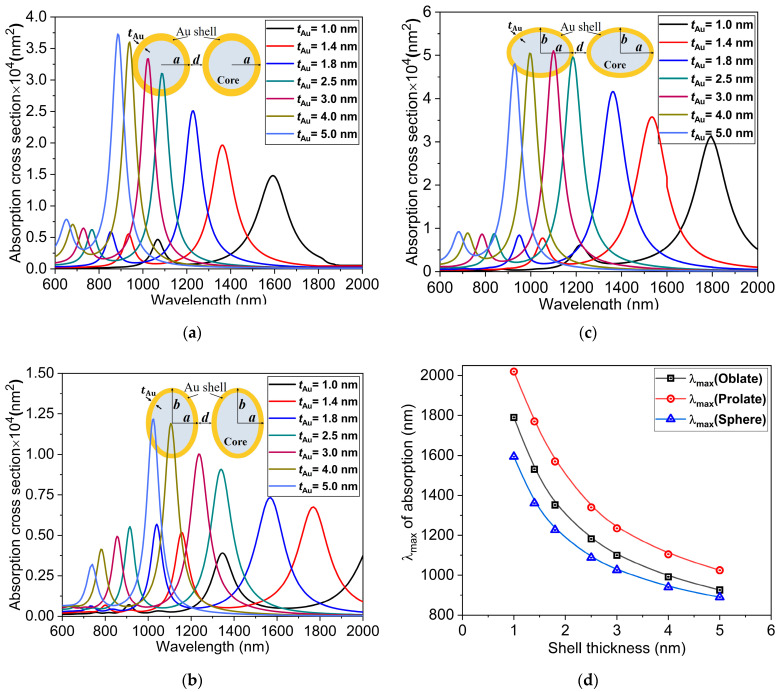
Absorption cross sections at different Au shell thicknesses for the (**a**) spherical, (**b**) prolate and (**c**) oblate Au core-shell nanostructures. (**d**) Variations of the LSPR peak position $(\lambda_{\max})\;$ with the Au shell thickness for the spherical $\left(r=20\;\mathrm{nm},\;\;d=1\;\mathrm{nm}\right)\;$ and non-spherical $\left(r_{\mathrm{eff}}=23\;\mathrm{nm},\;d=1\;\mathrm{nm}\right)$ Au core-shell nanostructures.

To further investigate the structure-dependent absorption characteristics of the spherical and non-spherical gold nanostructures, the thickness of the Au shell is considered as a parameter in this subsection. Figure 7 presents the absorption cross-sections at different shell thicknesses for the spherical and non-spherical dimer nanostructures while keeping the effective radius of the nanostructures and distance of separation fixed at $r_{\mathrm{eff}}=23\;\mathrm{nm}$ and d=1 nm. In order to see the effect of shell thickness, seven different shell thicknesses, ranging from 1 nm to 5 nm, are selected. The most distinctive feature in the three types of dimer nanostructures is that as the thickness of Au decreases, there is a clear red-shift in the LSPR peaks in the NIR-II window. This obvious red-shift with decreasing thickness can be attributed to an increase in the Au–Au shell interaction, which results in a greater energy separation between higher and lower energy modes [60]. For a shell thickness of 1 nm, the maximum red-shift for the spherical dimer nanostructure is 1591 nm, whereas those for the prolate and oblate nanostructures are 2095 nm and 1794 nm, respectively. The LSPR peaks of the lower energy modes in the longer wavelength region are mainly due to the dipole modes, and the higher energy modes occurring in the shorter wavelength region are due to the plasmon resonant coupling of quadrupole modes. A strong red-shift in the absorption peak for the lower energy resonance occurring in the longer wavelength region corresponds to the plasmon hybridization in the hollow core and outer shell of the dimer nanostructures. This plasmon hybridization in the Au nanostructures gives rise to the antisymmetrically coupled higher energy (antibonding) plasmon mode and a symmetrically coupled lower energy (bonding) plasmon mode [61].

**Figure 8:**
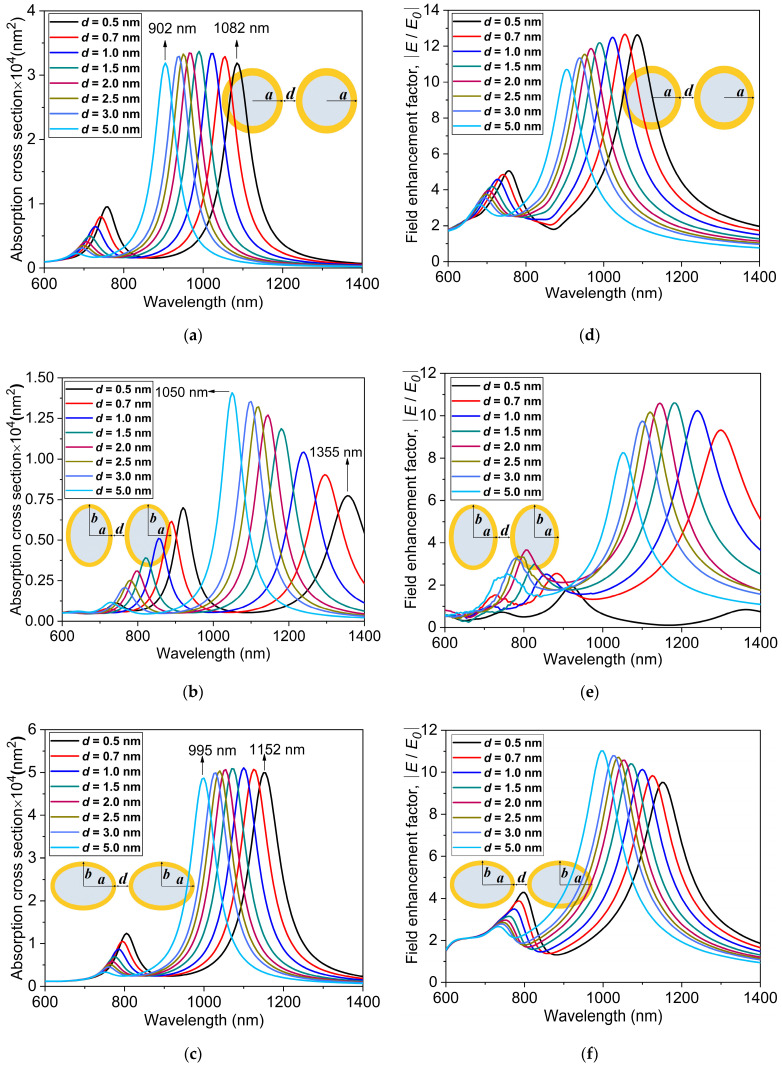
Absorption cross-sections at different distances of separation $\left(d=0.5\;\mathrm{nm}\;\mathrm{to}\;5\;\mathrm{nm}\right)$ for the (**a**) spherical nanostructure$\;\left(r=20\;\mathrm{nm},\;t_{\mathrm{Au}}=3\;\mathrm{nm}\right)$, (**b**) prolate, and (**c**) oblate Au core-shell dimer nanostructures $\left(r_{\mathrm{eff}}=23\;\mathrm{nm},\;\eta =3\right).$ The electric field enhancement factors, $\left|E/E_{0}\right|$, for the (**d**) spherical, (**e**) prolate, and (**f**) oblate nanostructures.

The evolution of LSPR in the core-shell dimer nanostructures with spherical and non-spherical shapes is numerically investigated and depicted in Figure 8.

An experimental investigation on the AuNSs using water as the inner core and surrounding material was performed previously [26]. Here, we study the effect of the separation distance $\left(d\right)$ between the outer surfaces of spherical$\;\left(r=20\;\mathrm{nm},\;t_{\mathrm{Au}}=3\;\mathrm{nm}\right)$ and non-spherical$\;\left(r_{\mathrm{eff}}=23\;\mathrm{nm},\;\eta =3\right)$ dimer nanostructures. The total effective radius$\;(r_{\mathrm{eff}})$ for the prolate and oblate structures is kept the same as the outer radius of the spherical AuNS. Figure 8a–c shows the absorption cross-sections for the spherical, prolate, and oblate Au nanostructures, respectively. As the separation distance between the nanoshells decreases from d=5 nm to 0.5 nm, there is a red-shift in the absorption cross-section peak for the three nanostructures. A significantly stronger interaction of the individual nanostructures due to the dimer configuration with a much smaller distance of separation gives rise to a larger red-shift in the absorption cross-section peak. The most red-shift into the NIR-II window occurs for the least separation distance d=0.5 nm with $\lambda_{\max}$ equal to 1082 nm, 1355 nm, and 1152 nm for the spherical, prolate, and oblate Au nanostructures, respectively. In particular, two distinct resonance peaks are observed in the absorption spectra, one in the longer wavelength region (NIR-II window) and the other in the shorter wavelength region (NIR-I window), as can be seen in Figure 8a–c. The resonance peaks with higher magnitudes in the longer wavelength region occur due to the coupling of the bonding modes in the dimer structure, whereas the peaks with very low magnitudes in the shorter wavelength region are a result of the coupling of the anti-bonding modes of the individual spherical and non-spherical nanostructures [62]. As the distance of separation decreases from d=5 nm to 0.5 nm, the higher energy quadrupole modes in the shorter wavelength region are red-shifted by a small amount, which is in good agreement with previous studies [63,64].

For the same aspect ratio η=3, the peaks of absorption cross-sections are above $4\times 10^{4}\;{\mathrm{nm}}^{2}$ for the oblate nanostructure, whereas they are well below$\;2\times 10^{4}\;{\mathrm{nm}}^{2}$ for the prolate structures, suggesting that the absorption of the oblate structure is more than twice as large as that of the prolate structure. Interestingly, for the oblate and spherical nanostructures, the peak of absorption is barely influenced by a change in the distance of separation. For the prolate structure, on the other hand, the peak shows greater dependence on the distance of separation and, with increasing distance, drastically increases ($1.40\times 10^{4}\;{\mathrm{nm}}^{2}$ for d=5 nm to $0.77\times 10^{4}\;{\mathrm{nm}}^{2}$ for d=0.5 nm). This dependency and resulting red-shift are due to the strong surface plasmon resonance coupling, indicating a significant electromagnetic interaction between the dimer nanostructures for the distances of separation considered in this study. Figure 8d–f shows the electric field enhancement values,$\;\left|E/E_{0}\right|$, for the spherical, prolate, and oblate Au dimer nanostructures, respectively. As can be seen in the figures, as the distance of separation increases, the blue-shift in the LSPR peak is observed. For the spherical nanostructure, as the distance of separation increases from 0.5 to 5 nm (see Figure 8f), the electric field enhancement undergoes a slight decrease and the LSPR peak shifts nearly 175 nm towards the shorter wavelength (1085 nm for d=0.5 nm to 910 nm for d=5 nm).

## 4. Conclusions

In this paper, we presented a numerical analysis of the absorption cross-sections and electromagnetic field enhancement of the spherical and non-spherical core-shell dimer nanostructures by solving the Helmholtz equation, using the complex dielectric function of gold derived from the Drude–Lorentz model. The study is performed by introducing finite element modelling in COMSOL Multiphysics with dimer nanostructures encapsulated by perfectly matched layers (PMLs) and scattering type boundary conditions. The present numerical model is validated by comparing the numerically obtained absorption cross-sections with those of Oldenburg et al. [52]. Upon coupling of two core-shell nanoparticles to form a dimer, the localized surface plasmon resonance (LSPR) peaks are rigorously red-shifted, specifically providing an extensive range of tunability in the NIR-II window. The monomer counterpart with the identical morphology does not exhibit this behaviour since the range of tunability is limited to the NIR-I window. Furthermore, when compared to the monomer counterparts of gold nanoshell (AuNS), the relative shift in the peak position for dimer nanostructures clearly displays superior sensitivity. In addition, the absorption cross-sections and electric field enhancement at different refractive indices of the surrounding medium, gold shell thicknesses, and distances of separation between the nanostructures are discussed in this paper. The slopes of the relative shift in the peak position for the three dimer nanostructures indicate that the prolate dimers are the most sensitive to change in the refractive index of the surrounding medium, with a wide range of LSPR tunability in the NIR-II biological window. The study shows that the LSPR peak position is influenced more by the thickness of the Au shell than the distance of separation between the nanostructures. The results reported here can be crucial in biomedical applications for accurately predicting the optical properties of gold nanostructures.

## Data Availability

The data presented in this study are available on request from the corresponding author.
